# Candy cane revision after Roux-en-Y gastric bypass

**DOI:** 10.1007/s00464-019-06988-4

**Published:** 2019-08-08

**Authors:** Anna Kamocka, Emma Rose McGlone, Belén Pérez-Pevida, Krishna Moorthy, Sherif Hakky, Christos Tsironis, Harvinder Chahal, Alexander Dimitri Miras, Tricia Tan, Sanjay Purkayastha, Ahmed Rashid Ahmed

**Affiliations:** 1 Department of Metabolism, Digestion and Reproduction, Hammersmith Hospital, Imperial College London, 6th Floor Commonwealth Building, Du Cane Road, London, W12 0NN UK; 2 Department of Surgery and Cancer, Imperial College London, London, UK; 3 Bariatric Surgical Unit at the Imperial Weight Centre, Imperial College NHS Healthcare Trust, London, UK; 4 Department of General Surgery, Cairo University, Giza, Egypt

**Keywords:** Candy cane, Hockey stick, Roux-en-Y gastric bypass complications, Revisional surgery

## Abstract

**Background:**

An excessively long-blind end of the alimentary limb following a Roux-en-Y gastric bypass (RYGB), known as a ‘candy cane’ (CC), may cause symptoms including abdominal pain, regurgitation and vomiting. Very few studies have examined the efficacy of surgical resection of the CC.

**Objectives:**

The aim of this study was to assess sensitivity of preoperative diagnostic tools for CC, as well as perioperative outcomes and symptom resolution after CC revision surgery.

**Setting:**

High volume bariatric centre of excellence, United Kingdom.

**Methods:**

Observational study of CC revisions from 2010 to 2017.

**Results:**

Twenty-eight CC revision cases were identified (mean age 45 ± 9 years, female preponderance 9:1). Presenting symptoms were abdominal pain (86%), regurgitation/vomiting (43%), suboptimal weight loss (36%) and acid reflux (21%). Preoperative tests provided correct diagnosis in 63% of barium contrast swallows, 50% of upper gastrointestinal endoscopies and 29% computed tomographies. Patients presenting with pain had significantly higher CC size as compared with pain-free group (4.2 vs. 2 cm, *p* = 0.001). Perioperative complications occurred in 25% of cases. Complete or partial symptom resolution was documented in 73% of patients undergoing CC revision. Highest success rates were recorded in the regurgitation/vomiting group (67%).

**Conclusion:**

Surgical revision of CC is associated with good symptom resolution in the majority of patients, especially those presenting with regurgitation/vomiting. However, it carries certain risk of complications. CC diagnosis may frequently be missed; hence more than one diagnostic tool should be considered when investigating symptomatic patients after RYGB.

Bariatric or metabolic surgery is a highly efficient mode of treatment for obesity and related conditions. As the number of patients with obesity soars [1], there is an increasing demand for bariatric surgery. There were over 21,000 bariatric operations in 2015–2017 in the United Kingdom alone [2]. The benefits extend far beyond weight loss, as bariatric surgery is also associated with type 2 diabetes (T2D) remission and amelioration of diseases including hypertension, hypercholesterolaemia and obstructive sleep apnoea [3, 4]. Furthermore, bariatric surgery is safe, with low mortality and morbidity rates reported by large registries including the National Bariatric Surgery Registry in the United Kingdom [2], International Federation for the Surgery of Obesity and Metabolic Disorders (IFSO) Global Registry [5] and Scandinavian Obesity Registry (SOReg) [6]. Laparoscopic Roux-en-Y gastric bypass (RYGB), considered by many a gold-standard bariatric procedure, accounts for the majority (54.1%) of bariatric procedures performed worldwide [5].

Nonetheless, bariatric surgery carries a risk of early and late postoperative complications [7]. Post-surgery patients may present with diverse symptoms including abdominal pain, nausea, regurgitation or vomiting and poor weight loss or weight regain, and it can be difficult to determine the underlying cause. Some of these symptoms have been associated with presence of a ‘candy cane’ (CC), also referred to as a ‘hockey stick’. This anatomical phenomenon has been defined as an excessively long-blind end of the alimentary limb proximal to gastrojejunostomy which can occur after a Roux-en-Y gastric bypass (RYGB) [8–10]. The aetiology of CC formation has not been fully established but the possible causes are a progressive dilatation of the blind end of the alimentary limb or leaving an excessively long-blind end of the afferent limb during gastrojejunostomy formation during RYGB. It can be diagnosed through an upper gastrointestinal endoscopy, radiological imaging such as barium contrast swallow (Fig. 1), computed tomography (CT) or intraoperatively.

**Fig. 1:**
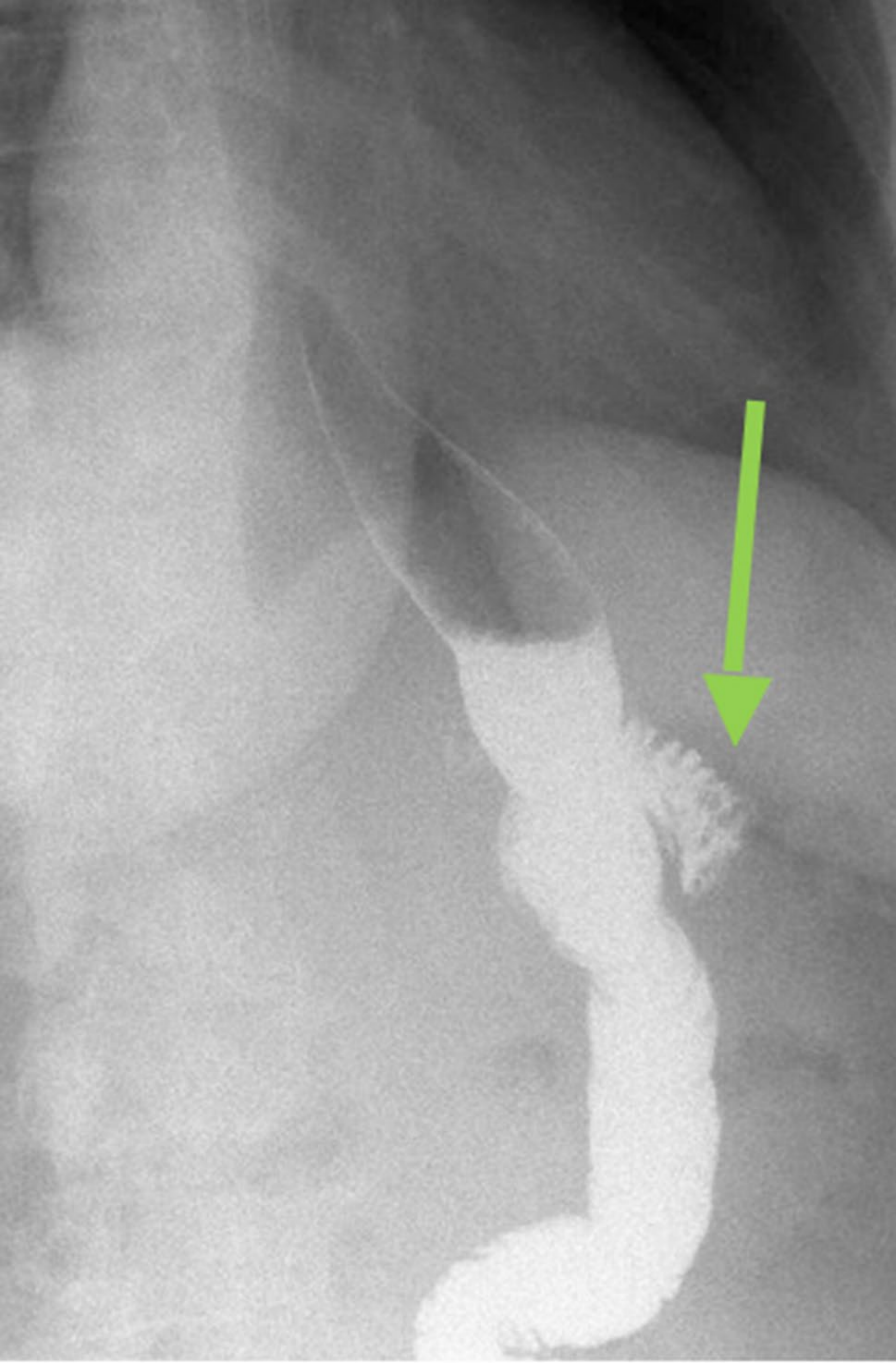
Candy cane demonstrated on a barium swallow (arrow)

There are very few studies (mainly small volume case series) that have investigated the efficacy of CC resection in alleviation of symptoms listed above [8–10]. Therefore, we have reviewed our experience in managing patients with CC after RYGB. The aims of our study were to assess diagnostic accuracy of commonly used investigations for the presence of candy cane, determine safety profile of CC revision surgery and record short-term outcomes.

## Methods

From our prospectively collated bariatric surgery database, we selected all cases of CC surgically revised between 2010 and 2017 at our high case volume bariatric centre of excellence. All operations were performed laparoscopically and involved a diagnostic laparoscopy to exclude any other pathologies, including internal herniae. Following that, resection of the CC segment was performed using a linear stapler with a 34-Fr orogastric tube passed through the gastrojejunostomy in order to avoid narrowing it. No revision of gastrojejunostomy was required. Length of the CC was assessed intraoperatively with reference to the laparoscopic instruments, which is a standard technique at our centre. In all cases where mesenteric and/or Petersen’s spaces were identified as open, laparoscopic closure was performed. Cases where another simultaneous procedure was performed (such as cholecystectomy, internal hernia repair, major adhesiolysis or a minimiser ring placement) were excluded from the study in order to avoid confounding factors interfering with CC revision outcomes review. All barium contrast swallow tests and CT scans were reported by radiologists, and diagnosis of CC was made by assessing the redundant blind-end loop and measuring its length. All upper gastrointestinal endoscopies were performed and reported by experienced gastroenterologists. Data were collected from patient’s medical notes and where necessary were supplemented by telephone follow-up with patients directly.

Variables analysed included demographic data, pre- and post-CC revision weight, Body Mass Index (BMI), indications for CC revision, perioperative and follow-up data, symptom resolution and complications. Suboptimal weight loss was defined as failure to achieve 20% total body weight loss (TBWL) following primary RYGB or weight regain of 25% or more. Complications were classified according to the Clavien–Dindo classification [11].

Statistical analysis with SPSS v.20 was performed. Data where values were normally distributed are presented as mean ± SD as well as ranges and percentages. Descriptive statistics were computed for all variables. Chi-square test was performed for comparison between the CC size and different symptom categories (e.g. pain, regurgitation/vomiting). Student’s *t* test was used for analysis of continuous data. All *p* values are two-sided. In order to test the accuracy of the diagnostic test in correctly detecting CC, the area under the ROC Curve was calculated. The values can range from 0.5 (no diagnostic ability) to 1.0 (perfect diagnostic ability), where 0.7 to 0.8 is considered acceptable and over 0.8 is considered excellent [12].

Study was performed as part of routine clinical practice and audit of hospital outcomes; therefore, ethics committee approval was not required. Data collection was performed in compliance with the Declaration of Helsinki.

## Results

### Patient characteristics

**Fig. 2:**
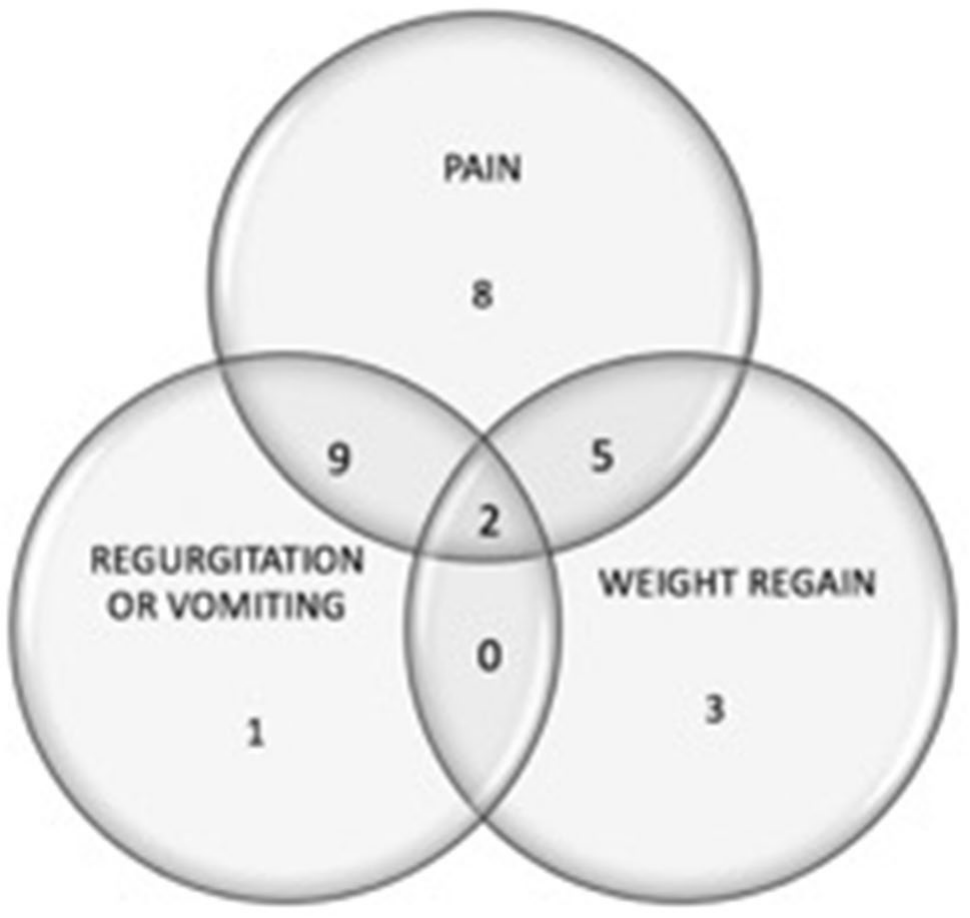
Most common indications for candy cane revisional surgery

Twenty-eight patients underwent surgical revision of CC. Characteristics of the study population are presented in Table 1. Ten patients who presented with suboptimal weight loss reported either a mean 7 ± 11% TBWL following primary RYGB (range 4% regain to 18% TBWL) or 40 ± 11% weight regain from nadir weight (27–57%). The most common indication for candy cane revision was pain (24 patients; 86%) (Table 2), which was postprandial in 14 patients (58%). The majority of patients (16; 57%) presented with more than one symptom (Fig. 2).

**Table 1:** Baseline cohort characteristics

Variable	Mean ± SD (range)/percentage (*n* number)/median (range)
Number of participants	28
Age	45 ± 9 years (24–60)
Gender	89% female (25)
Weight at primary RYGB BMI at primary RYGB	118 ± 16 kg (92–154) 46 ± 7 kg/m^2^ (33–58)
Total body weight loss after primary RYGB	26 ± 12% (4% regain to 50% TBWL)
Weight prior to CC revision BMI prior to CC revision	87 ± 17 kg (58–141) 34 ± 7 kg/m^2^ (23–54)
Median time between primary RYGB and CC revision	3 years (0.6–7)

**Table 2:** Preoperative symptoms and their resolution after CC surgery

	Preoperatively % (*n*)	Resolution % (*n*)^a^
Pain	86 (24)	57 (13)
Regurgitation or vomiting	43 (12)	67 (8)
Reflux	21 (6)	60 (3)

^a^Percentage experiencing resolution or improvement of those that presented with the symptom pre-revision (one patient was lost to follow-up)

### Preoperative investigations

**Fig. 3:**
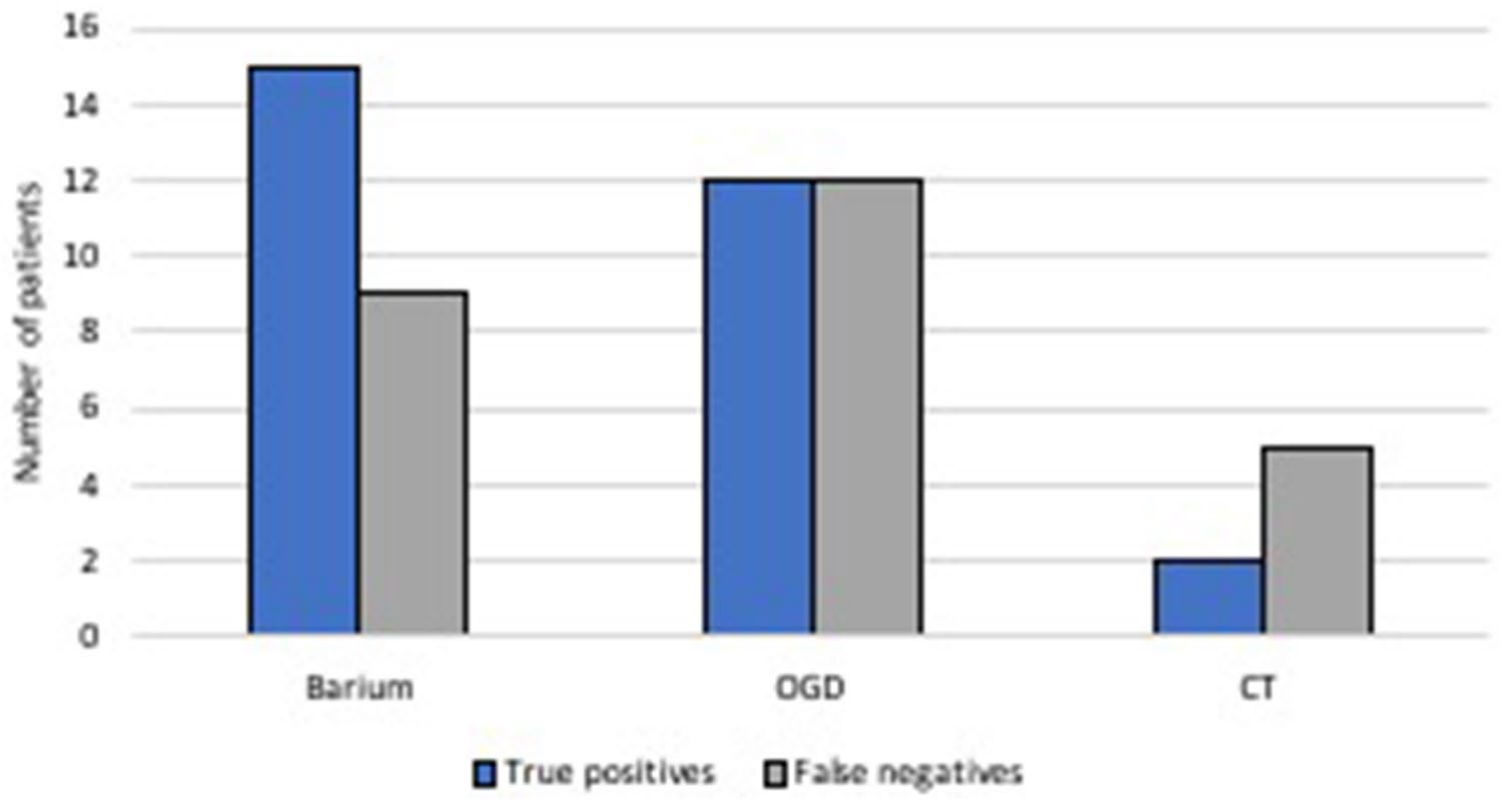
Diagnostic accuracy of preoperative investigations

All patients were investigated preoperatively with one or more of diagnostic tools including barium contrast swallow (24 patients; 86%), upper gastrointestinal endoscopy (24; 86%) and/or CT (7; 25%). When assessing their sensitivity, barium swallow detected accurately CC in 63% of cases (15 of 24) and it had the greatest accuracy in CC diagnosis with area under ROC curve of 0.705. Endoscopy had a diagnostic true positive rate of 50% (12 of 24), whereas CT had a true positive rate of 29% (2 of 7) (Fig. 3). There were two cases where both gastroscopy and barium swallow were reported as negative (one of these patients was found to have a small (2 cm) CC intraoperatively and in the second case CC size was not specified).

Size of the CC was quantified in 21 patients (75%) and its length ranged from 2 to 10 cm (4.0 ± 1.9 cm). Barium meal test was able to diagnose > 90% of the CC over a cut-off point of 2.5 cm.

### Perioperative outcomes

Mesenteric and/or Petersen’s spaces closure was performed in 54% (15 of 28 patients). Most CC revision operations were performed as day-case procedures (82%) with a median length of stay of 0 days (0–5). Within 30 days after the CC revision, there were 7 complications recorded (25%). As per Clavien–Dindo classification [11], 1 patient had Grade 1 complication (constipation and abdominal pain), 2 had Grade 2 complications (2 port site wound infections) and further 4 were classified as Grade 3b (bowel enterotomy on port insertion, haematoma from bleeding lumbar branch, intraabdominal collection drained laparoscopically, anastomotic ulcer at gastrojejunostomy). There were no readmissions to the hospital within the 30 days postoperatively and complications were managed either during the same admission or in the ambulatory care. There were no mortalities.

### Postoperative outcomes

Twenty-seven patients attended at least one follow-up consultation (96%). Post-revision clinical follow-up was sustained for 1.3 ± 1.3 years (2 months to 5 years).

At the end of the follow-up period, 7 (27%) patients had complete symptoms resolution and 12 (46%) reported resolution or improvement in at least one of the symptoms. The remaining 7 patients (27%) did not report any change postoperatively. When presenting complaints were analysed separately, the highest rate of symptom resolution was achieved in patients who had presented with regurgitation and/or vomiting (67%) (Table 2). Of the 10 patients who presented with suboptimal weight loss pre-revision surgery, 1 was lost to follow-up and 3 (33%) reported moderate weight loss post-CC revision of 13 ± 4% (9–18%) of TBWL. Of the 18 patients that did not present pre-revision with suboptimal weight loss, 4 experienced weight loss of 15 ± 9% TBWL (7–26%) following CC revision. However, 3 patients from this group underwent CC revision within 1.5 year after the RYGB, so this outcome may still be attributable to an ongoing weight loss following primary procedure. Fourth patient was later diagnosed with a pharyngeal pouch which could have led to weight loss as a result of regurgitation and vomiting.

Of 7 patients who did not have symptoms resolution following CC revision, all remained under the care of bariatric multidisciplinary team and underwent subsequent follow-up investigations, depending on presenting symptoms (barium swallow, CT, upper GI endoscopy, abdominal ultrasound, repeat laparoscopy, hydrogen breath test, colonic transit study and/or colonoscopy). Of these, 5 patients were later diagnosed with other pathologies: small bowel bacterial overgrowth, pharyngeal pouch, slow transit colon (2 patients) and fibromyalgia. Further 2 patients remained under medical and psychology teams care for chronic pain management. One further patient, whose reflux symptoms partially improved after the CC surgery, was later diagnosed with oesophageal dysmotility (hypotensive lower oesophageal sphincter and frequent failed swallows).

### Associations between CC size, symptoms and outcomes

Patients presenting with pain had significantly higher CC size than the pain-free group (Table 3). No correlation was found between the CC size and weight regain or any of the gastrointestinal symptoms listed above. Furthermore, there was no association between CC size and symptoms resolution.

**Table 3:** Preoperative symptoms and CC size

Candy cane size	Asymptomatic CC size Mean ± SD	Symptomatic Mean ± SD	*p* value
Pain	2.0 ± 0.0	4.2 ± 2.0	< 0.001
Weight regain	4.2 ± 1.5	3.8 ± 2.2	0.558
Regurgitation or vomiting	3.9 ± 2.1	4.0 ± 1.4	0.948
Reflux	3.9 ± 2.1	4.0 ± 1.4	0.948

*SD* standard deviation

## Discussion

Although RYGB is a safe and effective operation, a proportion of patients will later undergo revision surgery [13]. Indications for revision surgery fall broadly into two camps: operation failure—predominantly patients who have experienced suboptimal weight loss or weight regain—and intractable symptoms, such as abdominal pain or vomiting. Such revision surgery is successful for some, but many patients will experience no benefit [14, 15].

Candy cane or hockey stick after RYGB refers to an excessively long-blind afferent Roux limb at the gastrojejunostomy site. This anatomical phenomenon, which may be detected on preoperative investigations or during subsequent surgery, has been attributed as a cause of both RYGB failure and a wide range of common postoperative symptoms and is in some centres considered an indication for revision surgery [8, 9]. Despite this, there are very little data regarding the efficacy of surgical treatment for the condition as well as diagnostic accuracy of preoperative investigations to reveal CC.

Our study is the largest series of surgically treated candy canes to date. Our data support previous case series which describe heterogeneous symptomatology of CC [8, 9]. A proportion of patients post-RYGB present with diverse symptoms and/or evidence of surgical failure: our data support the commonly held clinical belief that CC should be considered among the differential diagnoses in such cases. Our results show, however, that widely employed investigation methods have poor sensitivity for the diagnosis of CC. Therefore, failure to diagnose a candy cane preoperatively does not exclude the diagnosis. Barium swallow, which had an overall true positive rate of 63%, does become more sensitive when the size of the CC is 2.5 cm and above (> 90% true positive rate). Nonetheless, two or even three diagnostic investigations may be necessary in order to reveal CC in symptomatic patients after RYGB.

In our series of an unselected cohort of patients with CC, the vast majority experienced complete or partial symptom resolution (73%) following CC revision. The rate of symptom resolution reported by us is lower than 94% presented by Aryaie et al. in their series of 19 patients with preoperatively defined ‘CC syndrome’ [9]. This could be due to the fact that patients with very specific symptom profile, i.e. postprandial epigastric pain or nausea and vomiting were selected in that study, whereas we included patients with all presenting syndromes and their combinations. The patients who benefited most from the CC surgery in our study were those presenting with regurgitation and/or vomiting (67% symptom resolution rate). More than half of the patients complaining of abdominal pain or reflux benefited from the CC revision (57% and 60% symptom resolution rate, respectively). It has been hypothesised that these symptoms may occur due to decreased small bowel motility in the blind-end loop with associated dilatation from food residual and possibly a co-existing bacterial overgrowth [9]. Therefore, resection of the CC is expected to alleviate these symptoms early postoperatively.

It has been debated whether patients suffering from poor weight loss or post-RYGB weight regain would benefit from the CC revision. Our data showed weight loss in 3 of 10 patients who had presented with suboptimal weight lost post-RYGB and in further 4 patients who had not complained of poor weight loss or weight regain prior to the CC revision. However, 3 patients from the latter group underwent the revisional surgery within 1.5 year from the primary RYGB and further 1 patient was later diagnosed with a pharyngeal pouch. These factors could have contributed to the observed weight loss, which could have happened regardless of the revision surgery. Furthermore, the weight loss following the CC surgery could be influenced by the intensified multidisciplinary follow-up which these patients would have received. Other theories regarding weight loss after the CC revision are the relative narrowing of the gastrojejunostomy following the revision and the removal of the ‘food reservoir’ [9], but the influence of those is difficult to assess in this study setting.

All patients who did not report any improvement in their symptoms after the CC revision continued to be investigated and treated by the bariatric multidisciplinary team. In 5 cases, other diagnoses (such as small-intestine bacterial overgrowth) were found and further 2 patients received ongoing support for pain management. It is therefore not only important to investigate further patients in whom CC revision does not result in symptom resolution but also to consider additional preoperative investigations such as routine testing for small-intestine bacterial overgrowth (which is currently a routine practice in our department).

The rate of complications seen in this study is significant but it is comparable to those of other studies of revisional bariatric surgery [14–16]. Therefore, as with other revisional surgery, it is recommended that they are performed at large volume bariatric centres to minimise the risk.

We acknowledge that this study has limitations. Firstly, the cohort of patients studied was small and heterogeneous, especially with regard to the symptom profile, size of the CC and length of the postoperative follow-up. Nonetheless, this is the largest series to date. Secondly, we did not have a comparator group in this study. It is plausible that some patients may have experienced improvement in symptoms over time without the surgical intervention and enhanced multidisciplinary follow-up after revisional surgery may have played role in improved outcomes. Moreover, the number of patients in our series may have been too small to detect relationships between presenting symptomatology and the likelihood of symptom resolution post-revision. There was no standardised scoring for symptoms assessment pre- and post-CC revision which could have affected reported symptom resolution rate as it was based on patient’s report at the follow-up. It is also worth noting that due to the design of our study, we cannot make any conclusions regarding the specificity of the investigations assessed for CC detection. Lastly, with just over half of the patients undergoing a prophylactic closure of mesenteric and/or Petersen’s defects, it is possible that some might have had an undiagnosed internal hernia, and this potentially could be a confounding factor when interpreting postoperative symptom resolution.

Nonetheless, as the largest series of CC revisions to date, this study adds in a meaningful way to our understanding of this post-RYGB complication. Our data would suggest that even in the situation where a CC is newly diagnosed during a diagnostic laparoscopy, revision should be performed in symptomatic patients, giving them a high chance of at least partial symptomatic improvement.

## Conclusion

CC remains a significant challenge in terms of diagnosis and management. It is likely to be responsible for some cases of both primary RYGB failure as well as intractable symptoms post-RYGB. More than one diagnostic tool (barium swallow ± upper gastrointestinal endoscopy ± CT) is likely to be required when investigating symptomatic patients after RYGB. Revision of CC is associated with good rates of symptom resolution (especially regurgitation and/or vomiting) in the majority of patients.

## References

[1] Organisation WH. Obesity and overweight 2018 http://www.who.int/news-room/fact-sheets/detail/obesity-and-overweight. Accessed 02 Jan 2019

[2] National Bariatric Surgery Register 2017. http://www.bomss.org.uk/third-nbsr-report-preview/. Accessed 10 July 2018

[3] Miras AD, Kamocka A, Patel D, Dexter S, Finlay I, Hopkins JC (2018). Obesity surgery makes patients healthier and more functional: real world results from the United Kingdom National Bariatric Surgery Registry. Surg Obes Relat Dis.

[4] Sjostrom L, Gummesson A, Sjostrom CD, Narbro K, Peltonen M, Wedel H (2009). Effects of bariatric surgery on cancer incidence in obese patients in Sweden (Swedish Obese Subjects Study): a prospective, controlled intervention trial. Lancet Oncol.

[5] IFSO. IFSO Global Registry Report 2017 http://www.ifso.com/wp-content/themes/ypo-theme/pdfs/final-3rd-ifso-report-21st-august-2017.pdf. Accessed 02 Jan 2019

[6] SOReg. Annual Report SOReg 2014 Part 2 (2014) ) Follow-up weight changes, change in comorbidity, long-term complications and quality indicators on the clinical level. http://www.ucr.uu.se/soreg/in-english. Accessed 02 Jan 2019

[7] Greenstein AJ, O’Rourke RW (2011). Abdominal pain after gastric bypass: suspects and solutions. Am J Surg.

[8] Dallal RM, Cottam D (2007). “Candy cane” Roux syndrome—a possible complication after gastric bypass surgery. Surg Obes Relat Dis.

[9] Aryaie AH, Fayezizadeh M, Wen Y, Alshehri M, Abbas M, Khaitan L (2017). “Candy cane syndrome:” an underappreciated cause of abdominal pain and nausea after Roux-en-Y gastric bypass surgery. Surg Obes Relat Dis.

[10] Romero-Mejía C, Camacho-Aguilera JF, Paipilla-Monroy O (2010). “Candy cane” Roux syndrome in laparoscopic gastric by-pass. Cir Cir.

[11] Dindo D, Demartines N, Clavien PA (2004). Classification of surgical complications: a new proposal with evaluation in a cohort of 6336 patients and results of a survey. Ann Surg.

[12] Mandrekar JN (2010). Receiver operating characteristic curve in diagnostic test assessment. J Thorac Oncol.

[13] English WJ, DeMaria EJ, Brethauer SA, Mattar SG, Rosenthal RJ, Morton JM (2018). American Society for Metabolic and Bariatric Surgery estimation of metabolic and bariatric procedures performed in the United States in 2016. Surg Obes Relat Dis.

[14] Vij A, Malapan K, Tsai C-C, Hung K-C, Chang P-C, Huang C-K (2015). Worthy or not? Six-year experience of revisional bariatric surgery from an Asian center of excellence. Surg Obes Relat Dis.

[15] Brolin RE (2005). Long limb Roux en Y gastric bypass revisited. Surg Clin N Am.

[16] Fulton C, Sheppard C, Birch D, Karmali S, de Gara C (2017). A comparison of revisional and primary bariatric surgery. Can J Surg.

